# Accuracy Analysis of a Multi-View Stereo Approach for Phenotyping of Tomato Plants at the Organ Level

**DOI:** 10.3390/s150509651

**Published:** 2015-04-24

**Authors:** Johann Christian Rose, Stefan Paulus, Heiner Kuhlmann

**Affiliations:** Department of Geodesy, Institute of Geodesy and Geoinformation, University of Bonn, Nussallee 17, 53115 Bonn, Germany; E-Mails: paulus@igg.uni-bonn.de (S.P.); heiner.kuhlmann@uni-bonn.de (H.K.)

**Keywords:** SfM and MVS photogrammetry, close-up laser scanning, plant phenotyping, organ-level parameterization

## Abstract

Accessing a plant's 3D geometry has become of significant importance for phenotyping during the last few years. Close-up laser scanning is an established method to acquire 3D plant shapes in real time with high detail, but it is stationary and has high investment costs. 3D reconstruction from images using structure from motion (SfM) and multi-view stereo (MVS) is a flexible cost-effective method, but requires post-processing procedures. The aim of this study is to evaluate the potential measuring accuracy of an SfM- and MVS-based photogrammetric method for the task of organ-level plant phenotyping. For this, reference data are provided by a high-accuracy close-up laser scanner. Using both methods, point clouds of several tomato plants were reconstructed at six following days. The parameters leaf area, main stem height and convex hull of the complete plant were extracted from the 3D point clouds and compared to the reference data regarding accuracy and correlation. These parameters were chosen regarding the demands of current phenotyping scenarios. The study shows that the photogrammetric approach is highly suitable for the presented monitoring scenario, yielding high correlations to the reference measurements. This cost-effective 3D reconstruction method depicts an alternative to an expensive laser scanner in the studied scenarios with potential for automated procedures.

## 1. Introduction

With the rise of phenotyping, the demand for access to the plants' 3D shape has become of significant importance [1]. Various methods using laser scanning [2], time of flight cameras [3,4] or structured light approaches [5], opening the door to 3D phenotyping, have been published. Close-up laser scanning has shown its advantage of high accuracy and high resolution [6] combined with direct access to the point cloud, but requires huge investment costs (100 k€ in advance. Using the 3D shape, descriptive parameters, like the plant volume or the geometry of single organs, can be extracted. A detailed parameterization and monitoring of growth or reactions to environmental changes are possible and can be tracked at the organ level. Furthermore, the effect of occlusion, a huge challenge in 2D phenotyping approaches [7,8], can be reduced tremendously.

In recent years, with the boost in computational power and the widespread availability of digital cameras, photogrammetric approaches, like structure from motion (SfM) [9] and multi-view stereo (MVS) [10], have found their way into phenotyping. These technologies offer non-invasive and non-destructive ways to measure a huge variety of plant characteristic traits through the creation of a full 3D point cloud of the plant. Simultaneously, they hold potential for future automatic measuring processes.

Ivanov *et al.* [11] were the first to use a photogrammetric approach to build a 3D point cloud of such a complex medium as a plant. They used a fixed base stereovision system pointing towards nadir, which was mounted 8.5 m above the ground to create a 3D model of the canopy of maize plants with a height of 2.5 m. From the model, they were able to derive the leaf position and orientation, as well as the leaf area distribution. This approach served as a stepping stone for future advances in the field of plant phenotyping with photogrammetric methods.

Aguilar *et al.* [12] used the close-range photogrammetric package, PhotoModeler Pro5 (Eos System Inc., Vancouver, BC, Canada), to measure the volume of several large tomato plants, as it is directly related to the Leaf Area Index (LAI), which represents the ground area covered by the plant canopy projected on the ground. The LAI itself is used for determining the optimal plant volume for pesticide application, as there is an ideal volume for spraying [13]. Around 400 target points of different colors attached to a plastic net were placed on each of the tomato plants, simulating the plants' enclosed surface. Afterwards, the target points were manually marked in PhotoModeler, and their 3D coordinates were calculated. Using these 3D coordinates, polygon meshes were computed, and from these, ultimately, the plant volume was calculated. A correlation of *R*^2^ = 0.75 between the measured volumes and manually-derived reference volumes was found, whereby the correlation between LAI and manual volume was found to be *R*^2^ = 0.817.

Santos *et al.* [14] used a consumer-grade camera mounted on a tripod to take images of basil and Ixora plants. The images were processed in an SfM approach called Bundler [15] to compute the camera's intrinsic parameters and relative poses [16] in order to compute sparse 3D point clouds of the plants. A patch-based MVS (PMVS) approach [17] was utilized to densify the sparse point cloud using the relative camera poses from the SfM model. Leafs and nodes were automatically segmented and classified afterwards [18]. They could show that these methods were able to effectively reconstruct the full three-dimensional shape of plants with a sparse canopy.

Paulus *et al.* [5] investigated the applicability of several low-cost 3D imaging systems for the task of plant phenotyping. The Microsoft Kinect and the DAVIDlaser scanner were compared to the high-precision close-up Perceptron v5 laser scanner, which served as a reference method. From the volumetric shape of sugar beet taproots, their leaves and the shape of wheat ears, plant parameters were extracted and compared to the reference measurements. They showed that low-cost sensors were as suitable as high-cost sensors, like the Perceptron v5, depending on the parameter of interest and the required level of detail.

Jay *et al.* [19] used SIFT keypoints to find homologous points between overlapping image regions. They applied an SfM approach named MicMac (MicMac 2007, IGN, Paris, France) to compute 3D point clouds from sunflowers, Savoy cabbages, cauliflowers and Brussels sprouts organized in crop rows. Their motivation was to assess the applicability of the image-based SfM approach for high-throughput phenotyping in outdoor scenarios where a variety of uncontrolled factors, like leaf movement through wind and inhomogeneous lightening conditions, complicate the data acquisition. Image acquisition followed the classical remote sensing method in that the camera is pointed towards nadir and linearly moved along a translation axis alongside the crop rows. Images are taken regularly after a certain baseline length between successive images has been reached. After the point cloud construction, plant and background were separated using criteria, like height and the Excess Green Index. Afterwards, plant height and leaf area were measured and compared to reference measurements, yielding high correlations for every species.

As the application of the SfM and MVS 3D reconstruction concept for plant phenotyping grows, it is important to gain knowledge about the potential measuring accuracy for parameter extraction. Accuracy evaluation then requires a high number of reference measurements, which possess higher accuracy and resolution than the SfM/MVS measurements. In the present study, the commercial photogrammetric software, Pix4DMapper (Pix4D SA, 1015 Lausanne, Switzerland), based on SfM and MVS techniques, was used to non-invasively and non-destructively monitor the growth process of a set of tomato plants for a six-day period. Colored 3D point clouds of the plants were reconstructed solely from image sets taken with the DSLR camera Canon EOS 450D. From the point clouds, several plant parameters at the organ level important for phenotyping were extracted. For accuracy and error evaluation, reference data for the same tomato plants were created using the high accuracy close-up triangulation line scanner, Perceptron v5 (Perceptron Scan Works V5, Perceptron Inc., Plymouth, MI, USA), combined with an articulated measuring arm (Romer Infinite 2.0 (1.4 m), Hexagon Metrology Services Ltd., London, U.K.). Reconstructing highly detailed and accurate 3D point clouds, the Perceptron has been found to be a high-precision non-invasive phenotyping tool [6]. This is the first time a photogrammetric method has been compared to a reference method at this high level of detail and accuracy. The applicability of the photogrammetric approach is evaluated using the means of linear regression and the RMSE (root-mean-square-error) and MAPE (mean-absolute-percentage-error) indicators.

## 2. Method

### 2.1. Experimental Setup

Five three-week-old tomato plants were monitored over a period of 6 days, resulting in 30 point clouds per method. The BBCHscale [20] value, determining the developmental stage of the plants, was 14/15 at the beginning of the monitoring and reached 17/18 at the end of monitoring.

The plants were stored in a greenhouse for optimal nourishment. In each case, the plant itself grew in a nourishment cube of a standardized size (100 × 100 × 100 mm) made from rock wool. To reduce plant movement due to wind and to avoid measurement uncertainties due to high temperature and humidity, the plants were moved to a prepared measurement table outside the greenhouse. Direct sunlight radiation was minimized by window coverages, and the temperature was modest at about 20–25 °C. A joint in the table ensured an identical positioning and orientation of the plant throughout the monitoring period.

### 2.2. Reference Data through High-Accuracy Laser Scanning

**Figure 1:**
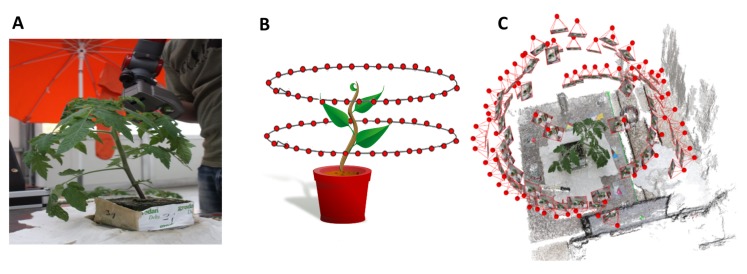
Both reconstruction methods: scanning the plant with the Perceptron v5 (**A**); the paths of the camera during image acquisition (**B**); the photogrammetric 3D reconstruction of the plant and the environment (**C**). Estimated camera positions are marked as red dots with attached image thumbnails.

The reference data were acquired using the articulated measuring arm Romer Infinite 2.0 combined with the close-up triangulation line scanner Perceptron v5. The system provides a point-to-point resolution of 14 μm with an accuracy of 45 μm. The measuring field has a depth of 110 mm and a mean width of 105 mm. This scanning combination has shown its applicability for 3D imaging of various plants, like grapevine [21], barley [6] and sugar beet [5]. The scanning field was manually moved over the plant (Figure 1A), resulting in a huge amount of single 3D scan lines. These were automatically combined to an occlusion-free and very dense point cloud of the plant. A point cloud consisted of 3–10 million points, dependent on the size and developmental stage of the plant. Data acquisition took about 10–15 min per plant.

### 2.3. Structure from Motion and Multi-View Stereo Using Pix4DMapper

The camera used for the image acquisition was a Canon EOS 450D (Canon Inc., Tokyo, Japan). It has a CMOS sensor with a pixel count of 12.2 MP and a dimension of 22.2 mm × 14.8 mm. The focal length is about 18 mm. The photogrammetric software Pix4DMapper was used to derive the 3D point clouds of the tomato plants. Pix4DMapper is based on SfM and MVS techniques employing keypoint matching [22,23] and bundle block adjustment. Processing runs semi-automatically with a working capacity of up to 1000 images. The PC used for processing worked with a Win7 64-bit system with 8 GB of memory and 8 cores of 3.7 GHz.

The quality and detail of the point cloud (Figure 1C) is determined by image quality and image content. To produce sharp edges without any image artifacts, the aperture, focus and exposure time were set in a balanced way to attain the largest depth of field possible without introducing motion blur. Room lights were switched on to reach an optimal image brightness. The heterogeneous texture of image content is an important prerequisite for keypoint detection and correct keypoint matching, the success of which determines the quality of the camera calibration and relative image orientation. Several texture-rich objects were placed around the table to increase the heterogeneity of the scene.

The images were taken by hand, *i.e.*, without a tripod, from a standing position using an image dimension of 3088 × 2056 pixels. To compensate for occlusions, two camera angles and heights were utilized. The camera was first held at a tilt of about 45° downwards from the horizontal at a height of about 50 cm over the top point of the plant. Focus was manually set and kept fixed. The depth of focus was increased by a small aperture to take sharp images. The user moved around the plant in a circular fashion, taking a new image every time the image content between the former and the current image had reached an overlap of about 70%. The whole time, the tilt of the camera and the initial distance of 50 cm were maintained as well as possible. The image overlap was estimated using the camera's viewfinder. Once the full circle had been closed, a second image set was taken in the same way with a different angle and height. The camera angle was horizontal and at a height at about the middle height level of the plant (Figure 1B). Again, the full circle around the plant was closed.

In this way, about 40–70 images per plant were recorded. During the monitoring phase, the plants grew about 10–15 cm in height while their canopy developed significantly, as well. Image acquisition took about 2–3 min per plant, regardless of the plants' growth stadium, as the amount of images necessary to image the whole plant did not change drastically. This corresponds to a mean acquisition speed of about one image every three seconds. The image sets were separately processed with Pix4DMapper, yielding colored 3D point clouds of the plant and close-by objects (Figure 1C). A point cloud consisted of about 15 k points at the beginning and of about 55 k points at the end of monitoring.

### 2.4. Point Cloud Preparation

Ahead of the parameter derivation, the point clouds of both sensors have to be processed. The preparation procedures and the methods of parameter derivation will be described in the following chapter. The point clouds were processed using Geomagic Studio 12 (Raindrop Geomagic Inc., Morrisville, NC, USA).

The laser scanning point clouds were cleaned from outliers manually. The point clouds were rasterized, resulting in a point-to-point distance of 0.5 mm to achieve a homogenous point distribution. Irrelevant objects were manually removed.

**Figure 2:**
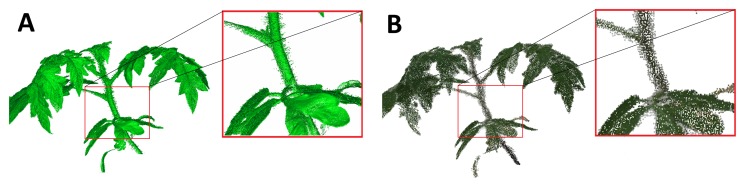
(**A**) A shaded and artificially-colored laser scanning point cloud; (**B**) a photogrammetric point cloud in real colors. The difference in point density is clearly visible.

Noise reduction and surface smoothing filters were applied to the Pix4dMapper point clouds in a way that geometrical features remained sharp and features of smaller scale, like tiny branches or leaves, were maintained. The point clouds were visually controlled for missing parts (e.g., from occlusions) after computation. Figure 2 shows a laser and a Pix4Dmapper point cloud in their entirety before preparation.

**Figure 3:**
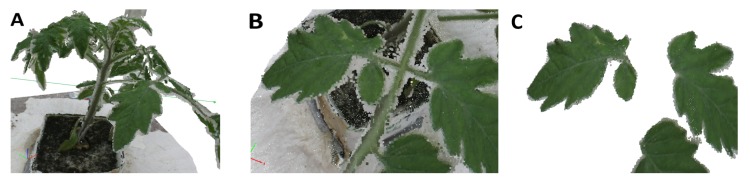
Close-up view of a photogrammetric point cloud before (**A,B**) and after (**C**) preparation (leaves only). The point size was enhanced for visualization.

Remaining outliers and false object points clearly distinguished through their texture (white and grey points at leaf and stem borders; Figure 3) were manually removed. Irrelevant objects visible in the point clouds, e.g., the table and nourishment cube, were manually removed until only the plant itself remained.

Initially, the Pix4DMapper point clouds are arbitrarily scaled. They were thus manually scaled to units of millimeters. The metric scaling factor was derived through the known value of a geometrical feature in the point cloud, e.g., the length of an object, and its real millimeter value. We either used the measuring table's length or width in millimeters, as at least one side was always fully visible in the point cloud. The scale factor is the ratio of the feature in millimeters and in the pixel system of the original point cloud. The original point cloud coordinates were multiplied with the deduced scale factor to reach a scaling in millimeters. In this way, an individual scale factor was determined for every point cloud.

### 2.5. Parameter Extraction

Four parameters were extracted from the point clouds: single leaf areas, cumulated leaf area, main stem height and volume of the convex hull of the whole plant. Stem height and leaf areas were derived using Geomagic, while the convex hull was calculated using MATLAB 2009b routines (The MathWorks Inc., Natick, MA, USA). The parameter extraction was identical for both methods.

Main Stem Height: The height of the main stem is an important indicator for the growth response of plants after fertilizer application [24] or under varying CO_2_ rates in the environment [25]. In this study, the beginning of the main stem was defined as the point where the stem emerges from the plant cube. The end was defined as the point of intersection of the first lateral branch after the two cotyledon leaves—the first leaves the seedling develops after emerging from the soil for nourishment—and the stem. For height determination, points covering the area from the beginning to the end of the main stem were selected, and a least-square approximation for a cylinder was applied, as it represents a standard approach in phenotyping [26,27]. The height of the estimated cylinder was taken as the main stem height.

Leaf area: The analysis of the leaf area is important in determining the plant's developmental stage [28], chlorophyll production [29] and health status. Furthermore, it serves as an indicator for mechanical tasks, such as pesticide application [12]. Stem and branch points were manually cut from the plant model, so that only leaf points remained. These were meshed, whereby the plausibility of the meshed surface was visually verified by its smoothness. The sum of the meshed areas constitutes the cumulated leaf area of the plant. Furthermore, 26 single leaf areas from two different plants were extracted to assess how accurately the approach is able to measure single leaves. Attention was focused on selecting leaves of different sizes (small, medium, large), forms and crookedness to assess its general applicability.

Convex hull: The convex hull is defined as the shape of an object created through joining its outermost points. From 2D images, the convex hull is often used to estimate the surface coverage of plants [30,31]. In 3D, it is used in the close-up range, e.g., in [32] to determine the soil exploration extent of root systems. It further serves as an estimator for tree canopy biomass [33] and has been used as an effective indicator for plant drought on barley plants [6].

Tables 1 and 2 illustrate the influence of the cleaning process on the final results. Especially, the leaf area estimation is affected by false object points on leaf borders.

### 2.6. Estimation of Accuracy and Error Distribution

Accuracy assessment is done by calculating the correlation coefficient *R*^2^ between laser and photogrammetric measurements through the means of linear regression. The RMSE (root-mean-square-error) and MAPE (mean-absolute-percentage-error) indicators are used for error estimation. They are calculated using Equations (1) and (2), whereby *ref* designates the reference measurement from the Perceptron v5 and *act* refers to the actual measurement extracted from the photogrammetric point clouds:

(1) $$\text{RMSE}=\sqrt{\text{mean}{\left(\text{ref}-\text{act}\right)}^{2}}$$

(2) $$\text{MAPE}=\text{mean}\left(\left|\frac{\text{ref}-\text{act}}{\text{ref}}*100\right|\right)$$

## 3. Results

**Figure 4:**
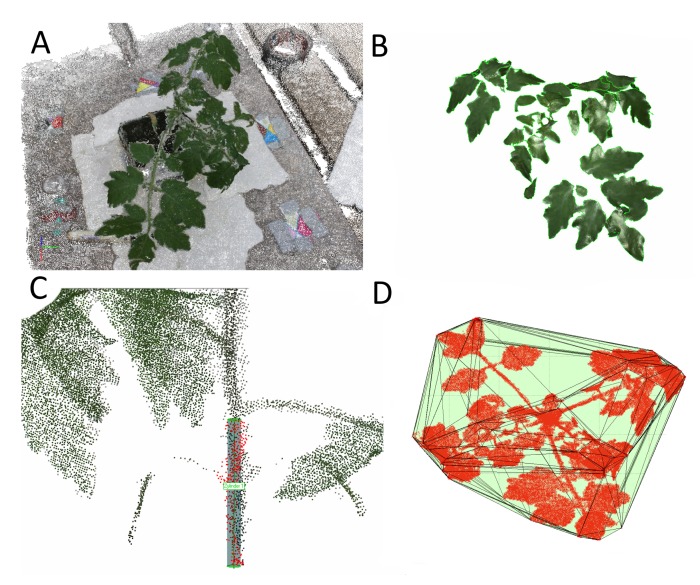
(**A**) The whole plant; (**B**) meshed leaves separated from stems and branches; (**C**) the measuring of the stem height; and (**D**) the computed 3D convex hull encompassing the plant.

All plants were reconstructed in their entirety through the usage of the two complementary perspective angles during data acquisition (Figure 2B; Figure 3A) with only very filigree parts missing in some cases (Figure 4C). Fine leaf veins are clearly distinguished texturally from the rest of the leaf.

**Figure 5:**
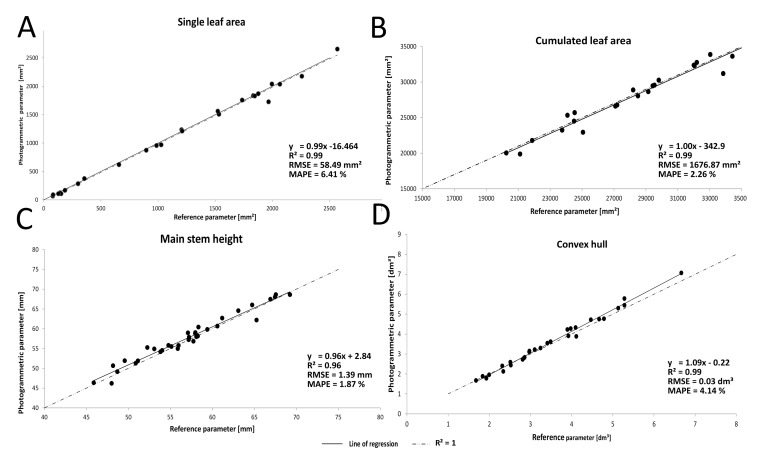
Regression analysis, RSME and MAPE results for the extracted parameters. (**A**) Single leaf area; (**B**) cumulated leaf area; (**C**) main stem height; (**D**) convex hull.

At the leaf and branch borders, triangulation errors were noticed (grey points at leaf and branch borders; Figure 3B). All parameters extracted from the Pix4DMapper point clouds yielded high correlations of *R*^2^ ≥ 0.96 to the parameters from the reference point clouds. The parameter single leaf area (Figure 5A) yielded a correlation *R*^2^ = 0.99 to the reference measurements. The RMSE reached 58.49 mm^2^, while the MAPE lay at 6.41%. The cumulated leaf area (Figure 5B) yielded a correlation of *R*^2^ = 0.99. The RSME for the cumulated leaf area reached 1679 mm^2^. The MAPE lay at 2.26%. A correlation of *R*^2^ = 0.96 was reached for the main stem height (Figure 5C) with an RMSE of about 1.39 mm and an MAPE lying at 1.87%. The convex hull (Figure 5D) yielded a correlation of *R*^2^ = 0.99, with an RMSE reaching 0.03 dm^3^ and an MAPE lying at 4.14%.

**Figure 6:**
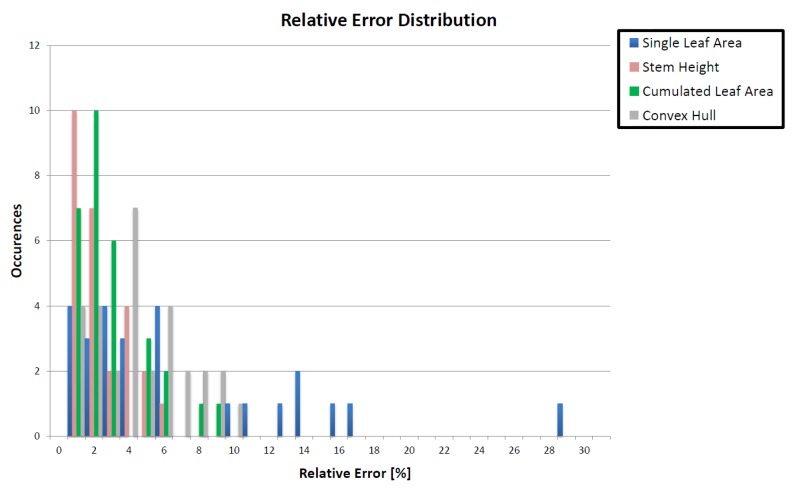
Histogram of the parameters' relative error distribution.

The relative error between reference and actual measurements mostly remained below 10% for all parameters. Relative errors over 10% were present only for single leaf area measurements (see Section 4). Figure 6 shows a histogram of the relative error distributions of all parameters.

## 4. Discussion

The results show that the SfM-/MVS-based method is well suited for determining the parameters inspected here. All extracted parameters highly correlate with the reference measurements, yielding a coefficient of *R*^2^ lying between 0.96–0.99. The MAPE lay under 7% for all parameters, thus lying under the limit of tolerance of 10%. This error limit was defined in [34] as the error already inherent between manual measurements. Furthermore, according to [34], detecting morphological changes in the plant between different measuring dates with a MAPE under this limit is still possible.

Measuring crooked and curled leaves is possible. Reconstructing small leaves in their completeness depends on their visibility in multiple images, as well as the level of detail and sharpness of the images. Additionally, small leaves are more sensible to filtering and, thus, more prone to loss of true area. This is seen in Figure 6, where the single leaf area alone from all parameters bears relative errors over 10% up to 30%. The level of detail can be increased by lowering the distance of the camera, while sharpness can be achieved by a wide depth of field. The plausibility of the meshed area was accounted for through visual inspection, but should be replaced by mathematical smoothness constraints. Holes in meshed areas can be filled manually and automatically if deemed plausible.

The stem height was measured manually using a cylindrical best-fit primitive, which allowed measuring even when the stem was only partly reconstructed. The stems beginning and end are defined by visual cues alone. A mathematical definition of the stems' beginning and end is needed to make measuring more objective. Furthermore, as the stem shape usually does not grow completely straight, the cylindrical form can only approximate its shape and height. More precise ways of parameterization need to be investigated, e.g., through the approximation of multiple adjacent cylinders or through circle approximation.

The convex hull is computed using the full plant without being separated into single plant organs first. As such, the convex hull is effortlessly extractable through automatic methods to monitor growth processes and reaction to environmental conditions, such as drought [6].

Data acquisition was done manually with a user taking images by hand without the use of a tripod, making the approach very adaptable and space-saving. Expert knowledge for the data acquisition is not necessary. The SfM approach calculates the cameras intrinsic parameters automatically and, in this way, avoids the long camera calibration phase before acquisition often necessary for stereo systems, as used in [35]. It is a very quick in the field method taking 2–3 min for data acquisition per plant. The Perceptron v5 is superior in point resolution and accuracy, but the time needed for data acquisition increases with plant size and complexity due to the limited working distance of 110 mm. The here depicted 3D reconstruction method is a cost-effective method amounting to 6500 € for the commercial Pix4DMapper software and to about 2500 € for a high-end camera. In comparison, the laser scanning system amounts to about 100 k€. We stress that while a specific commercial software was used for the point cloud derivation, at least similar if not equal results could probably be achieved by using alternative solutions, as demonstrated in [10], where the general accuracy and completeness of six MVS algorithms was investigated on several challenging 3D objects. Alternative software, like Bundler/PMVS (see e.g., [14]) or Agisoft Photoscan [36] are potential candidates. Likewise, open source software, like CloudCompare [37] and Octave [38] pose well-suited alternatives for parameter computation, point removal and scaling procedures.

A difficulty is the post-processing character, as the completeness and the quality of the point cloud can only be assessed after computation. During the data acquisition, it is therefore important to take images with sufficient content overlap and from complementary perspectives to avoid occlusion and to reconstruct the plant completely. False white and grey object points predominantly exist on leaf and stem borders. These are probably due to false matches caused by the homogeneous texture of the white foil of the nourishment cube and white paper towels placed underneath the cube. These could be removed through color filtering.

The amount of time invested per plant in post-processing procedures amounts to 10 to 20 min for point cloud generation, 5 min for manual scaling and 5–10 min for error removal and separation into plant organs. Data acquisition using the Perceptron laser scanner amounted to 10–15 min in contrast. However, as the working distance of the scanner is only 110 mm, the time needed for a full scanning will grow with the plant's size and canopy complexity. Images, on the other hand, capture more parts of the plant in a shorter period of time. There will be a plant size for which scanning the object is no longer feasible, and SfM/MVS methods require less time for data acquisition and 3D reconstruction. In [18,21], the feasibility of automatic geometry-based segmentation and classification algorithms for plant organ parameterization was already demonstrated. These could further be improved through the integration of the color information of the photogrammetric point clouds. In combination with methods for automatic coordinate system transformation of the point cloud (e.g., image geotagging, usage of measuring arms) and color filters for error removal, these approaches could reduce the need for supervised post-processing and enhance the amount of throughput that phenotyping often requires [1]. The color information should further be utilized for detecting plant reactions to environmental stress factors, like drought.

Future works should focus on extracting additional morphological parameters, like LAI, leaf length/inclination and stem diameter, which can probably be extracted from the point clouds with a similar accuracy. The limit of detectable details should be studied, as well. While only one kind of plant was used, the morphology of the plants changed drastically over the period of monitoring. In this period, they grew from a relatively open and simple canopy with some larger leaves, into a complex and denser canopy with varying leaf sizes and branch thicknesses. Our results demonstrate that the reconstruction is possible even under changing plant morphologies. The approach should nevertheless be tested on plants with even more complex and denser morphologies.

## 5. Conclusions

We show that the here presented photogrammetric approach depicts a reliable tool for high accuracy phenotyping at the organ level. A correlation of *R*^2^ ≥ 0.96 to high accuracy reference data was reached. The data acquisition is very simple and can be handled by non-trained personal, while the approaches flexibility holds potential for application in a variety of scenarios. It thus poses a suitable alternative to expensive and stationary tools for the studied scenarios.

## Figures and Tables

**Table 1:** Example of the influence of cleaning on the cumulated leaf area.

	**Cumulated Leaf Area (mm^2^)**		

	**Pre-Cleaning**	**After Cleaning**	**Difference (%)**
Pix4DMapper	50,795.9	48,468.1	**4.6**
Laser (Reference)	48,661.2	48,404.1	**0.5**

**Table 2:** Example of the influence of cleaning on the convex hull.

	**Convex Hull (dm^3^)**		

	**Pre-Cleaning**	**After Cleaning**	**Difference (%)**
Pix4DMapper	5.301	5.217	**1.6**
Laser (Reference)	5.131	5.125	**0.1**
